# Plasma Glucosylsphingosine in *GBA1* Mutation Carriers with and without Parkinson's Disease

**DOI:** 10.1002/mds.28846

**Published:** 2021-11-06

**Authors:** Matthew Surface, Manisha Balwani, Cheryl Waters, Alexander Haimovich, Ziv Gan‐Or, Karen S. Marder, Tammy Hsieh, Linxia Song, Shalini Padmanabhan, Frank Hsieh, Kalpana M. Merchant, Roy N. Alcalay

**Affiliations:** ^1^ Department of Neurology, College of Physicians and Surgeons Columbia University New York New York USA; ^2^ Department of Genetics and Genomic Sciences Icahn School of Medicine at Mount Sinai New York New York USA; ^3^ The Neuro (Montreal Neurological Institute‐Hospital) McGill University Montreal Quebec Canada; ^4^ Department of Neurology and Neurosurgery McGill University Montreal Quebec Canada; ^5^ Department of Human Genetics McGill University Montreal Quebec Canada; ^6^ Nextcea, Inc. Woburn Massachusetts USA; ^7^ The Michael J. Fox Foundation for Parkinson's Research New York New York USA; ^8^ Northwestern University Feinberg School of Medicine Chicago Illinois USA

**Keywords:** Parkinson's, Gaucher's, glucocerebrosidase, lipidomics

## Abstract

**Background:**

Biallelic mutations in the *GBA1* gene encoding glucocerebrosidase cause Gaucher's disease, whereas heterozygous carriers are at risk for Parkinson's disease (PD). Glucosylsphingosine is a clinically meaningful biomarker of Gaucher's disease but could not be assayed previously in heterozygous *GBA1* carriers.

**Objective:**

The aim of this study was to assess plasma glucosylsphingosine levels in *GBA1* N370S carriers with and without PD.

**Methods:**

Glucosylsphingosine, glucosylceramide, and four other lipids were quantified in plasma from N370S heterozygotes with (n = 20) or without (n = 20) PD, healthy controls (n = 20), idiopathic PD (n = 20), and four N370S homozygotes (positive controls; Gaucher's/PD) using quantitative ultra‐performance liquid chromatography tandem mass spectrometry.

**Results:**

Plasma glucosylsphingosine was significantly higher in N370S heterozygotes compared with noncarriers, independent of disease status. As expected, Gaucher's/PD cases showed increases in both glucocerebrosidase substrates, glucosylsphingosine and glucosylceramide.

**Conclusions:**

Plasma glucosylsphingosine accumulation in N370S heterozygotes shown in this study opens up its future assessment as a clinically meaningful biomarker of *GBA1*‐PD. © 2021 The Authors. *Movement Disorders* published by Wiley Periodicals LLC on behalf of International Parkinson Movement Disorder Society.

The *GBA1* gene encodes the lysosomal enzyme, acid β‐glucocerebrosidase (GCase; EC 3.2.1.45). Biallelic mutations in *GBA1* cause the lysosomal lipid storage disorder, Gaucher's disease (GD).^1^ A common coding variant of *GBA1*, N370S, is pathogenic for GD and represents one of the most common genetic risk factors for Parkinson's disease (PD) in the heterozygous carriers,^2^, ^3^, ^4^ but the mechanism underlying this increased risk for PD remains unknown.

Pathogenic variants in *GBA1*, including N370S, decrease GCase activity. GCase metabolizes glycosphingolipids, a class of lipids essential for membrane structure and a range of cellular functions. The prevailing hypothesis for PD associated with *GBA1* mutations (ie, *GBA*‐PD) is that a decrease in GCase activity alters the lipid composition of membranes and results in increased α‐synuclein aggregation and accumulation in the lysosomes.^5^ The two key substrates of GCase are glucosylceramide (GluCer) and glucosylsphingosine (GluSph). Recent studies suggest that GluCer and GluSph accumulation may promote α‐synuclein aggregation,^6^, ^7^, ^8^ further indicating the importance of studying these lipids as biomarkers of *GBA*‐PD. However, accumulation of GluCer or GluSph in *GBA*‐PD brain or cerebrospinal fluid (CSF) has not been unequivocally demonstrated,^9^, ^10^, ^11^ posing a major challenge to the loss‐of‐function hypothesis for *GBA*‐PD.

GluCer is by far more abundant than GluSph and is shown to accumulate in macrophages and plasma of people with GD. However, it is not a clinically useful biomarker for GD.^12^ By contrast, plasma GluSph levels correlate with disease burden in GD,^13^ leading to its routine use as a biomarker for GD severity, as well as response to therapy.^14^ GluSph is also a key regulator of the immune system. Thus, it would be important to develop sensitive assays to monitor GluSph levels and determine its utility as a biomarker of *GBA*‐PD.

In this study, we measured GCase substrates, GluSph and GluCer, as well as the product, ceramide, and additional lipids (galactosyl ceramide, galactosyl sphingosine, and glucosyl cholesterol) as negative controls in a cohort of *GBA1* N370S mutation carriers and noncarriers with and without PD. We aimed to test whether GluSph levels were elevated in *GBA1* N370S mutation carriers and whether levels were associated with PD status. We hypothesized, based on the GD literature, that plasma GluSph would be a superior marker of reduced GCase activity in comparison with plasma GluCer levels.

## Materials and Methods

Participants in this study were participants of the Spot study, which was previously described.^15^ The Spot study is an ongoing biomarker recruitment effort at Columbia University Irving Medical Center and the Icahn School of Medicine at Mount Sinai (ISMMS), through which we have identified (and reported on) 218 carriers of various *GBA1* pathogenic variants, including 135 carriers with PD and 83 carriers without PD.^15^, ^16^ In brief, *GBA1* carriers with and without PD were recruited among Columbia University Irving Medical Center patients with PD and spouse controls, and *GBA1* carriers without PD were recruited among family members of patients with GD (ie, parents or children, who are by definition obligate carriers) at the Lysosomal Storage Diseases Program at ISMMS. Demographics, as well as the Unified Parkinson's Disease Rating Scale (UPDRS) score in the *on* state and the Montreal Cognitive Assessment score, were collected from all participants. To reduce heterogeneity, we included only *GBA1* N370S heterozygotes in this study (and not other *GBA1* pathogenic variants). We further excluded *LRRK2* G2019S mutation carriers. In addition, we included four samples of N370S homozygotes with PD, who also by definition have GD (ie, GD/PD). Two of the four GD/PD participants were treated with enzyme replacement therapy at the time of recruitment. A total of 10 mL of whole blood per subject was collected in Ethylenediaminetetraacetic acid (EDTA) tubes, which were centrifuged, and from which 1 mL plasma aliquots per subject were extracted within 60 minutes of collection. Samples were stored in a −80°C freezer until processing. All clinical study procedures were approved by the Columbia University Institutional Review Board (and ISMMS Institutional Review Board, if collected at Mount Sinai, NY, USA), and all participants signed informed consents. All samples were shipped to Nextcea (Woburn, MA, USA) on dry ice in one batch.

### Quantitative Ultraperformance Liquid Chromatography Tandem Mass Spectrometry Analyses

The bioanalytics were conducted by Nextcea, where scientists remained blinded to participants' PD status and genotype before analysis.

A multiplexed quantitative ultraperformance liquid chromatography tandem mass spectrometry method was used to simultaneously quantitate plasma ceramides (Cer d18:1/16:0, d18:1/18:0, d18:1/20:0, d18:1/22:0, d18:1/24:0, d18:1/24:1), GluCers (GluCer d18:1/16:0, d18:1/18:0, d18:1/22:0, d18:1/24:0, d18:1/24:1), galactosylceramides (GalCer d18:1/16:0, d18:1/18:0, d18:1/22:0, d18:1/24:0, d18:1/24:1), GluSph (GluSph 18:1), galactosylsphingosine (GalSph 18:1), and glucosylcholesterol. The GluSph (GluSph 18:1) was well separated from galactosylsphingosine (GalSph 18:1). Standard curves were prepared from related standards using a class‐based approach. Internal standards were used for each analyte reported. A SCIEX Triple Quad 7500 mass spectrometer was used in positive electrospray ionization mode for detection (SCIEX, Framingham, MA, USA). Injections were made using a Shimadzu Nexera XR UPLC (ultraperformance liquid chromatography) system (Shimadzu Scientific Instruments, Kyoto, Japan). The instruments were controlled by SCIEX OS 2.0 software.

The parameters for assay validation included 3‐day intra‐assay and interassay accuracy and precision, sensitivity (lower limit of quantitation [LLOQ]), specificity, carryover, recovery, matrix effect, dilution integrity, and stability in human plasma. The LLOQ for all lipids measured was 5 pg/mL. Interassay and intra‐assay coefficients of variation for all dilutions (including LLOQ) were ≤20%. The specificity of the GluSph versus GluCer assay was evaluated at the LLOQ in six different lots of human plasma with stable isotope‐labeled GluCer and GluSph, where we observed no interference.

### Calibration and Data Processing

The intensities of the analytes and internal standards were determined by integration of extracted ion peak areas using SCIEX OS 2.0 software. Calibration curves were prepared by plotting the peak area ratios for each analyte to internal standard versus concentration. The model for the calibration curves was linear with (1/*x* ^2^) weighting. The concentrations of all lipids were quantitated in nanograms per milliliter (ng/mL) of plasma.

### Statistical Analysis

**FIG. 1:**
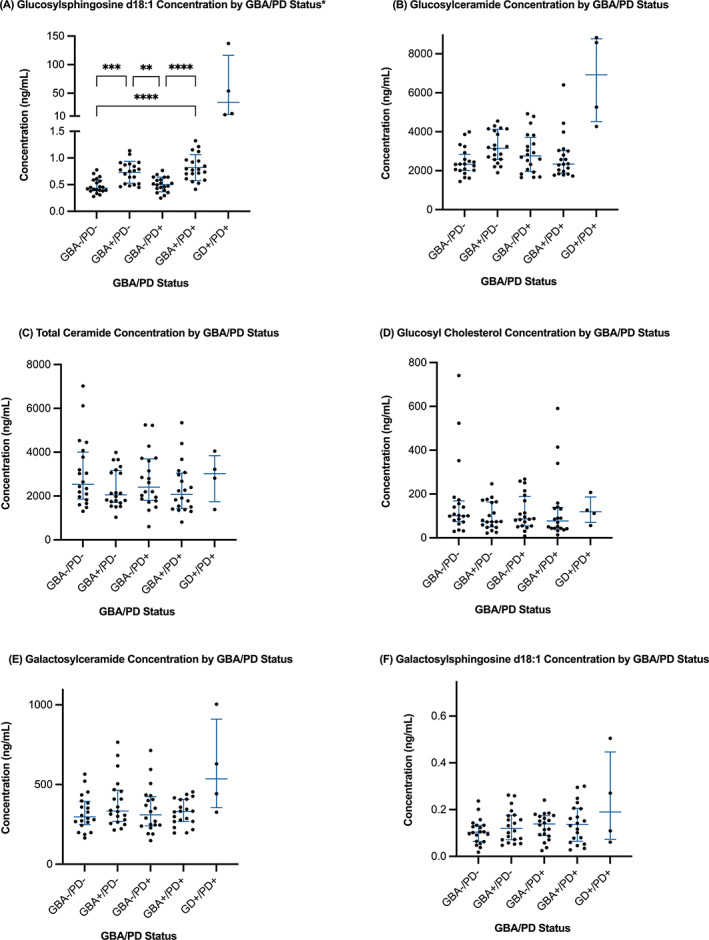
Plasma lipid concentration for glucolipids in *GBA* ^−^/PD^−^, *GBA* ^+^/PD^−^, *GBA* ^−^/PD^+^, *GBA* ^+^/PD^+^, and Gaucher's disease (GD)^+^/PD^+^, including: (**A**) glucosylsphingosine d18:1 (GluSph), (**B**) glucosylceramide (GluCer), (**C**) total ceramides, (**D**) glucosylcholesterol (GluChol), (**E**) galactosylceramide (GalCer), and (**F**) galactosylsphingosine d18:1 (GalSph). Patients with GD/PD had significantly higher plasma levels of GluSph and GluCer than all other groups (A and B). *GBA* ^+^/PD^−^ and *GBA* ^+^/PD^+^ had significantly higher GluSph levels than *GBA* ^−^/PD^−^ and *GBA* ^−^/PD^+^ (A: *GBA* ^−^/PD^−^ vs. *GBA* ^*+*^/PD^−^, *P* = 0.0002; *GBA* ^−^/PD^−^ vs. *GBA* ^+^/PD^+^, *P* < 0.0001; *GBA* ^−^/PD^+^ vs. *GBA* ^*+*^/PD^−^, *P* = 0.0012; *GBA* ^−^/PD^+^ vs. *GBA* ^+^/PD^+^, *P* < 0.0001). **P < 0.005; ***P < 0.0005; ****P < 0.0001.

Demographics and disease characteristics were compared across the four groups based on PD status and genotype (*GBA* ^*−*^/PD^*−*^, *GBA* ^+^/PD^*−*^, *GBA* ^*−*^/PD^+^, *GBA* ^*+*^/PD^+^). Groups were a priori matched by sex and age (PD^*−*^ versus PD^+^). We then compared the lipid species concentrations across the four groups. We computed total concentrations of ceramides, GluCer and GalCer, by summing all fatty acid analogs for each species. Although primary analyses compared the four groups, we included the four GD/PD subjects (positive controls) in Figure 1 for illustrative purposes. We further constructed regression models to predict *GBA1* mutation status (outcome), including sex, age, PD status, and lipid concentrations as predictors. Analyses were computed in SPSS. Figure 1 (and post hoc Tukey analyses) was computed using GraphPad Prism version 9.1.1 for macOS, GraphPad Software (GraphPad, San Diego, CA, USA; https://www.graphpad.com).

## Results

### Demographic Comparisons

Demographic and disease characteristics of the four groups (*GBA* ^*−*^/PD^−^, *GBA* ^+^/PD^−^, *GBA* ^*−*^/PD^+^, *GBA* ^*+*^/PD^+^) are presented in Table 1. The groups were matched by sex and age (PD versus non‐PD), and the PD groups were also similar in age at onset, disease duration, education, Montreal Cognitive Assessment performance, levodopa‐equivalent daily dose, and Unified Parkinson's Disease Rating Scale, Part III scores. Figure 1 demonstrates the plasma concentration of total ceramides, total GluCer, and GluSph d18:1 in *GBA1* N370S heterozygotes and noncarriers with and without PD and in four patients with GD/PD. The plasma lipid concentrations across the four groups are presented in Table 1 and Supporting Information Table S1. Next, we performed logistic regression models to test the association between *GBA1* status and plasma lipid concentrations, including age, sex, PD status, and the six lipid groups tested. GluSph was the only variable (and the only lipid) associated with *GBA* mutation status (*P* < 0.001).

**TABLE 1:** Demographics, disease characteristics, and lipid concentration data

	*GBA* ^−^/PD^−^	*GBA* ^+^/PD^−^	*GBA* ^−^/PD^+^	*GBA* ^+^/PD^+^	*P* value^a^
n	20	20	20	20	
Sex (male/female)	10/10	10/10	10/10	10/10	
Age, y	71.1 (9.1)	59.8 (9.2)	65.9 (12.8)	66.2 (9.5)	0.010
AAO	N/A	N/A	59.4 (14.2)	60.6 (9.6)	0.756
Education, y	16.6 (3.1)	18 (3.5)	16.4 (3.5)	16.4 (2.7)	0.329
MoCA score	26.6 (3.1)	26.5 (1.8)	24.9 (3.2)	25.1(2.9)	0.108
UPDRS III score			16.0 (8.3)	15.8 (7.7)	0.944
LEDD			593 (560)	447 (359)	0.332
Glucosylsphingosine d18:1	0.48 (0.14)	0.73 (0.20)	0.50 (0.14)	0.82 (0.24)	<0.001
Total glucosylceramides	2443.75 (706.45)	3217.22 (814.86)	2910.08 (1030.44)	2701.03 (1139.59)	0.071
Total ceramides	3038.51 (1554.18)	2338.91 (876.27)	2719.79 (1260.67)	2350.19 (1148.74)	0.228
Glucosylcholesterol	165.92 (178.04)	99.33 (63.37)	116.45 (80.43)	130.16 (148.91)	0.401
Total galactosylceramides	323.08 (110.55)	387.35 (154.74)	341.73 (144.46)	324.25 (81.96)	0.341
Galactosylsphingosine d18:1	0.11 (0.05)	0.13 (0.07)	0.13 (0.06)	0.14 (0.08)	0.417

Values are presented as mean (standard deviation). Data were analyzed by one‐way ANOVA. Participants were grouped by the presence or absence of either Parkinson's disease (PD^−^ or PD^+^) or the *GBA1* N370S mutation (*GBA* ^−^ or *GBA* ^+^).

^a^ *P* value represents overall effect by ANOVA.

AAO, age at motor onset; MoCA, Montreal Cognitive Assessment; UPDRS III, Unified Parkinson's Disease Rating Scale, Part III; LEDD, levodopa‐equivalent daily dose; N/A, not applicable.

## Discussion

The link between *GBA1* pathogenic variants and PD has opened a window of opportunity for multiple therapeutics targeting the GCase pathway. The mechanisms of interventions range from augmentation of GCase activity via chaperones or allosteric activators, inhibition of GluCer synthase, and *GBA* gene therapy.^17^, ^18^, ^19^ The therapeutic development trials require reliable biomarkers to establish target engagement/modulation and inform effective dose range. Furthermore, the penetrance of PD among carriers of pathogenic variants in *GBA1* is estimated at 10–30%, indicating that the majority of mutation carriers will never develop PD.^20^, ^21^, ^22^, ^23^ Thus, biomarkers that can prognosticate which *GBA1* pathogenic variant carriers are more likely to develop PD are required to conduct patient‐enriched trials.

To the best of our knowledge, this is the first study to successfully detect GluSph in the plasma of non‐GD *GBA1* mutation carriers. Importantly, our data demonstrate that a single allele of *GBA1* N370S is sufficient to result in a significant elevation in plasma GluSph levels. This effect was specific for GluSph among the panel of six lipids tested, including GluCer (Table 1, Fig. 1). The validity of our assays is also confirmed by the elevation in GluSph and GluCer in the four GD/PD cases. Thus, plasma GluSph offers a promising biomarker of target engagement/modulation in GCase‐targeted therapies for PD. However, it is possible (given our sample size) that we were underpowered to detect differences of smaller effect size, for example, in GluCer levels between carriers and noncarriers, or in GluSph levels between carriers with and without PD. Previous studies support the hypothesis that we were underpowered to identify small differences in GlucCer levels between carriers and noncarriers. Specifically, in the Parkinson's Progression Markers Initiative study, noncarriers had lower GluCer levels in CSF than carriers, but similar analysis in plasma (as done in this study) was not reported.^24^

It is important to note that GluSph, rather than GluCer, is emerging as a more disease‐relevant biomarker. A recent study reported higher levels of GluCer in the CSF in conjunction with a decrease in blood GCase activity in *GBA*‐PD compared with idiopathic PD, but the differences were observed primarily in those with severe *GBA* mutations (eg, L444P), but not the mild *GBA* mutation, N370S^11^ (GluSph was not assayed in this study). When GCase activity is deficient, the enzyme acid ceramidase metabolizes GluCer into GluSph.^25^ GluSph has been shown to cause oligomerization of α‐synuclein in vitro more efficiently than GluCer.^7^ More importantly, GluSph, and not GluCer, induced pathogenic templating of α‐synuclein in cells, including in induced pluripotent stem cell–derived neurons.^7^ Furthermore, a role of GluSph in immune cell activation is well documented,^26^ and GluSph levels in the brain correlated with neurological manifestations in GD.^27^ Because aberrant immune activation in PD is thought to contribute to pathogenesis, the elevation in plasma GluSph in N370S mutation carriers suggests that this lipid has the potential to be a disease pathophysiology marker even for prodromal *GBA*‐PD. However, the exact mechanism by which *GBA* mutations cause PD remains to be determined. For example, it is possible that reduced activity of GCase leads to lower intralysosomal levels of ceramide, which negatively affect the maturation and activation of cathepsin‐D.^28^, ^29^

We have not identified an association between plasma GluSph levels and PD status in the current samples of N370S carriers or with idiopathic PD. However, because GluSph levels were numerically higher in carriers with PD compared with carriers without PD (0.82 ± 0.24 versus 0.73 ± 0.20), and in noncarriers with PD compared with controls (0.50 ± 0.14 versus 0.48 ± 0.14), we posit that a larger cohort of *GBA* mutation carriers and noncarriers will be needed to investigate whether GluSph levels are associated PD status. Furthermore, future studies should include a wide range of *GBA1* pathogenic variants (eg, E326K) to test potential correlation between variant severity and GluSph levels. Studies that will examine GluSph levels in the brain and CSF in addition to blood plasma may firmly test its relevance as a disease marker, as is the case for GD.

In summary, these results indicate that plasma GluSph levels may be a useful target engagement/modulation biomarker for interventions aiming to increase GCase activity. Studies on larger cohorts would be required to test whether elevated GluSph levels are associated with PD risk or progression.

## Author Roles

Study concept and design: R.N.A., F.H., K.S.M., K.M.M., S.P.

Subject recruitment, assessment, genotyping, and blood collection: M.S., M.B., Z.G.‐O.

Biomarker assays: F.H., T.H., L.S.

Statistical analysis of data: R.N.A.

Initial drafting of manuscript: R.N.A., K.M.M., M.S., A.H.

Editing and approval of submitted manuscript: all authors.

## Financial Disclosures

Disclosures unrelated to the study: Manisha Balwani: research support (NIH/NIDDK, Genzyme, Alnylam, Mitsubishi‐Tanabe), honoraria (Genzyme, Takeda, Alnylam, Prevail, Freeline, Alexion). Cheryl Waters: research support (Biogen, Roche, Sanofi); consulting fees (Kyowa, Alexza, Sunovion); speaker's honoraria (Acadia, Acorda, Adamas, Amneal, Kyowa, Neurocrine, US WorldMeds). Ziv Gan‐Or: research support (The Michael J. Fox Foundation, Fonds de recherche du Québec–Santé, Canadian Consortium for Neurodegeneration in Aging, Canada First Research Excellence Fund through the Healthy Brain for Healthy Lives project), consulting fees (Denali, Inception Sciences [now Ventus], Idorsia, Lysosomal Therapeutics Inc., Prevail Therapeutics, Deerfield, Lighthouse, Neuron23, Ono Therapeutics, Handl Therapeutics, and Bial Biotech Inc.). Kalpana M. Merchant: research support (The Michael J. Fox Foundation), advisor/paid consultant (The Michael J. Fox Foundation, Caraway Therapeutics, Sinopia Biosciences, Nitrome, NuraBio, Retromer Therapeutics, Vanqua Biosciences). Roy N. Alcalay: research support (NIH, DoD, the Parkinson's Foundation, and The Michael. J. Fox Foundation), consultation fees (Avrobio, Caraway, GSK, Merck, Ono Therapeutics, and Sanofi).

## Supporting information


**Table S1** Concentrations of each lipid analyzed in carriers and non‐carriers with and without PD
Values are presented as mean (standard deviation). Data were analyzed by one‐way ANOVA. Participants were grouped by the presence or absence of either Parkinson's disease (PD− or PD+) or the GBA1 N370S mutation (GBA− or GBA+). ^a^ P value represents overall effect by ANOVA.

## Data Availability

The data that support the findings of this study are available on request from the corresponding author.
