# Supplementary material for: Plasma Glucosylsphingosine in GBA1 Mutation Carriers with and without Parkinson's Disease
Source: Mov Disord. 2021 Nov 6;37(2):416–21. doi: 10.1002/mds.28846 (PMC8840974; doi:10.1002/mds.28846)
Supplement: Supplementary file 1 — Table S1 Concentrations of each lipid analyzed in carriers and non‐carriers with and without PD Values are presented as mean (standard deviation). Data were analyzed by one‐way ANOVA. Participants were grouped by the presence or absence of either Parkinson's disease (PD− or PD+) or the GBA1 N370S mutation (GBA− or GBA+). a P value represents overall effect by ANOVA. [file MDS-37-416-s001.docx]

**Supplementary Table 1**

|  | **GD+PD**  **(n=4)** | ***GBA-*/PD-**  **(n=20)** | ***GBA*+/PD-**  **(n=20)** | ***GBA-*/PD+**  **(n=20)** | ***GBA*+/PD+**  **(n=20)** | ***P value* (excluding GD+PD)** |
| --- | --- | --- | --- | --- | --- | --- |
| **GluCer d18:1/16:0** | 1384.62 (479.61) | 665.07 (357.78) | 708.91 (352.16) | 696.06 (433.32) | 613.80 (310.64) | 0.849 |
| **GluCer d18:1/18:0** | 340.21 (136.82) | 147.98 (60.30) | 154.24 (54.25) | 156.53 (78.68) | 151.06 (88.86) | 0.983 |
| **GluCer d18:1/22:0** | 2828.84 (1236.90) | 865.29 (302.03) | 1305.58 (351.68) | 1189.16 (368.08) | 1092.04 (529.22) | 0.006 |
| **GluCer d18:1/24:1** | 2182.31 (892.89) | 765.41 (311.93) | 1048.49 (415.54) | 868.34 (383.59) | 844.15 (438.66) | 0.140 |
| **Cer d18:1/16:0** | 185.54 (48.74) | 189.80 (93.31) | 149.57 (53.67) | 174.49 (72.70) | 143.33 (65.93) | 0.159 |
| **Cer d18:1/18:0** | 65.95 (28.45) | 63.27 (38.40) | 46.67 (27.86) | 54.92 (28.12) | 46.00 (24.72) | 0.237 |
| **Cer d18:1/20:0** | 457.11 (218.59) | 514.50 (347.67) | 371.77 (200.29) | 411.90 (216.90) | 348.90 (208.38) | 0.172 |
| **Cer d18:1/22:0** | 651.06 (253.85) | 703.74 (368.60) | 508.07 (211.34) | 585.42 (240.50) | 527.93 (267.11) | 0.121 |
| **Cer d18:1/24:0** | 763.86 (292.91) | 841.75 (429.84) | 670.82 (278.90) | 822.38 (448.93) | 688.45 (313.73) | 0.347 |
| **Cer d18:1/24:1** | 744.03 (308.57) | 725.45 (331.93) | 592.02 (226.90) | 670.69 (317.29) | 595.59 (324.26) | 0.446 |
| **GalCer d18:1/16:0** | 65.57 (33.07) | 45.28 (17.05) | 53.28 (20.49) | 41.77 (19.41) | 48.24 (20.64) | 0.295 |
| **GalCer d18:1/18:0** | 31.02 (17.69) | 8.62 (6.92) | 13.52 (7.54) | 11.79 (7.40) | 11.18 (6.99) | 0.200 |
| **GalCer d18:1/22:0** | 267.50 (113.49) | 120.80 (39.00) | 154.10 (76.73) | 123.39 (47.24) | 125.60 (44.07) | 0.181 |
| **GalCer d18:1/24:1** | 236.35 (141.51) | 148.39 (96.16) | 166.44 (91.12) | 164.81 (98.69) | 139.24 (67.48) | 0.729 |

GD+PD: N370S homozygotes (Gauhcer Patients) with Parkinson’s disease. Cer = ceramide; GluCer = glucosylceramide; GalCer = galactosylceramide
